# Predictors of Mortality in Patients with Cardiac Device-Related Infective Endocarditis

**DOI:** 10.3390/tropicalmed9090193

**Published:** 2024-08-24

**Authors:** Gustavo Brandão Oliveira, Isabela Galizzi Fae, Vinícius Tostes Carvalho, Pedro Henrique Oliveira Murta Pinto, Roni Arley Silva Duque, Fernanda Alves Gelape, Fernanda Sophya Leite Cambraia, Guilherme Lelis Costa, Lucas Chaves Diamante, Renato Bráulio, Cláudio Léo Gelape, Marcos Roberto Sousa, Teresa Cristina Abreu Ferrari, Maria Carmo Pereira Nunes

**Affiliations:** 1 Programa de Pós-Graduação em Ciências Aplicadas à Saúde do Adulto, Faculdade de Medicina da Universidade Federal de Minas Gerais, Avenida Professor Alfredo Balena, 190, Santa Efigênia, Belo Horizonte 30130-100, MG, Brazil; brandaomed@gmail.com (G.B.O.); isabela.galizzi@gmail.com (I.G.F.); phmurta@gmail.com (P.H.O.M.P.); tferrari@medicina.ufmg.br (T.C.A.F.); 2 Departamento de Clínica Médica, Faculdade de Medicina da Universidade Federal de Minas Gerais, Avenida Professor Alfredo Balena, 190, Santa Efigênia, Belo Horizonte 30130-100, MG, Brazil; v-tostes@uol.com.br (V.T.C.); fernandacambraia.br@gmail.com (F.S.L.C.); guilherme_lelis_@hotmail.com (G.L.C.); lucasdiamante02@gmail.com (L.C.D.); 3 Hospital das Clínicas da Universidade Federal de Minas Gerais, Avenida Professor Alfredo Balena, 110, Santa Efigênia, Belo Horizonte 30130-100, MG, Brazil; roniduque@hotmail.com (R.A.S.D.); sousa.mr@uol.com.br (M.R.S.); 4 Faculdade de Ciências Médicas de Minas Gerais, Alameda Ezequiel Dias, 275, Centro, Belo Horizonte 30130-110, MG, Brazil; fernandagelape@hotmail.com; 5 Departamento de Cirurgia, Faculdade de Medicina da Universidade Federal de Minas Gerais, Avenida Professor Alfredo Balena, 190, Santa Efigênia, Belo Horizonte 30130-100, MG, Brazil; renatobraulio1000@gmail.com (R.B.); clgelape@uol.com.br (C.L.G.)

**Keywords:** infective endocarditis, implantable cardiac device, pacemaker, implantable cardioverter defibrillator, cardiac resynchronization therapy, prognosis, in-hospital mortality

## Abstract

Infective endocarditis (IE) associated with implantable cardiac devices (ICD) is a serious disease with high mortality rates. The increased number of ICD implants has led to increased ICD infection rates. The aim of this study was to characterize clinical, laboratory profiles and the prognosis of cardiac-device-related endocarditis (CDIE), as well as to identify predictors of in-hospital death. A total of 274 patients with IE were included in a prospective cohort (2007–2019). From these, 82 patients (30%) had CDIE (46 pacemakers, 23 cardioverter defibrillators, and 13 cardiac resynchronization therapy devices). Predisposed conditions; clinical, laboratory and echocardiographic parameters; etiologic agents; and in-hospital outcomes were evaluated. The mean age was 55.8 ± 16.4 years, where 64.6% were male. Among the clinical manifestations at diagnosis, the most prevalent were heart failure (67.9%), fever (60.5%), anorexia/hyporexia (44.4%), and heart murmur (37.5%). The median serum C-reactive protein (CRP) level at diagnosis was 63 mg/L (interquartile range [IQR] 20–161). Etiological agents were identified through positive blood cultures in 55% of cases. The main etiologic agents were negative-coagulase staphylococci (19.5%) and *Staphylococcus aureus* (18.3%). Vegetation was identified in 74 patients (90.1%). In-hospital mortality was 28%. CRP concentrations at diagnosis were identified as markers of disease severity (odds ratio [OR] 1.006; 95%CI 1.001–1.011; *p* = 0.016), and the worsening of heart failure was associated with unfavorable outcomes (OR 3.105; 95%CI 1.397–6.902; *p* = 0.005). Unlike what is traditionally accepted, CDIE does not have a better prognosis.

## 1. Introduction

Infective endocarditis (IE) is a severe disease associated with substantial morbidity and mortality [1,2]. It primarily affects the heart valves, the endocardial surface, or an indwelling cardiac device, often leading to complications including heart failure, stroke, and systemic embolism. Individuals at higher risk of IE include those with pre-existing heart conditions, particularly valvular heart disease, indwelling cardiac devices, intravenous drug users, and patients with a history of prior endocarditis. Despite improvements in diagnostic and therapeutic strategies over the last century, mortality rate have not significantly improved, remaining as high as 30% within one year [3,4,5,6,7]. The epidemiological profile of IE has changed in recent decades, with an increasing number of cases involving device-related infections now termed cardiac device–related endocarditis (CDIE). This differs from left-sided IE, which affects the native or prosthetic valves on the left side of the heart, including the mitral and aortic valves.

Cardiac implantable electronic devices are increasingly being used in cardiac disease management worldwide [8,9,10,11]. Infection is a major complication of cardiac device implantation, and it is associated with substantial morbidity, mortality, and healthcare costs [11]. The analysis of a population-based epidemiological study, conducted from 1998 to 2013, showed an increase in the frequency of patients with implanted pacemakers or defibrillators from 8.8% to 15.6%. During the same period, the prevalence of CDIE increased from 1.3% to 4.1% [12]. The absolute rate of device infection itself has recently increased, probably due to the increasing frequency of implantable cardioverter defibrillator (ICD) and cardiac resynchronization therapy (CRT) devices being implanted [10,13,14]. The incidence of IE is closely linked to the complexity of the device, and it is notably high during the first year after implantation [11].

The diagnosis of CDIE may be challenging owing to the broad spectrum of clinical manifestations and underlying conditions [8,15]. Symptoms and blood tests may be misleadingly normal, especially in the absence of lead vegetation, which does not rule out blood-stream infections [16]. Regardless of whether IE is directly associated with these devices, IE in itself is still a life-threatening disease with high mortality, especially in high-risk patients with cardiac devices [11]. Predictors of mortality in overall IE have indicated that complications during the in-hospital course, including embolic events, periannular extension of the infection, and valve dysfunction, leading to heart failure are the main factors associated with death [17,18,19]. In a previous study, we identified several predictors of mortality in a large contemporary cohort of patients with IE. These predictors include older age, C-reactive protein levels at hospital admission, vegetation length at diagnosis, and the development of heart failure or embolic events during antimicrobial therapy. These findings take into account patient characteristics and complications that arise during treatment [20]. As the population ages, comorbidity numbers increase, and indications of cardiac devices expand, the number of CDIE cases is expected to rise further. Therefore, the identification of patients at high risk for early mortality after CDIE diagnosis would permit a more aggressive therapeutic approach.

In the present study, we aimed to characterize the clinical and laboratory profile of patients with CDIE compared with other IE types with the purpose of determining the risk factors associated with mortality in the subset of patients with CDIE.

## 2. Materials and Methods

From 2007 to 2019, consecutive adult patients who were admitted to Hospital das Clínicas, Universidade Federal de Minas Gerais (UFMG), Belo Horizonte, Brazil (a tertiary-care university hospital), with definite or possible IE based on modified Duke criteria [21] were prospectively included in this study.

Demographic data, symptoms, cardiac murmurs, signs of heart failure, vascular and other immunologic phenomena, baseline comorbidities, and pre-existing cardiac condition were collected at hospital admission. Laboratory tests including complete blood count, C-reactive protein (CRP), serum chemistry, and urine analysis were recorded at baseline and during treatment.

Blood cultures were obtained from all patients before initiating antibiotic therapy. Cultures were also obtained from the excised fibrotic capsule of the device pocket and from the lead tip along with its attached fibrotic tissue at the time of device removal.

The Ethic Committee from UFMG approved the study protocol (ETIC 412/06), and written informed consent was obtained from all patients.

Infective endocarditis was categorized based on disease types as CDIE or left-sided IE, which includes native-valve or prosthetic-valve endocarditis on the left side of the heart.

The diagnosis of CDIE was suspected on clinical evidence of infection at the generator pocket including erythema, warmth, tenderness, fluctuance, wound dehiscence, erosion, or purulent drainage. Endocarditis was confirmed by the presence of vegetation on the tricuspid valve or at the end of the electrical lead in echocardiography or when the Duke criteria for IE were met [21]. A definitive diagnosis was based on the microorganisms detected by the cultures from the device pocket, as well as from electrode leads [9]. We also included cases of positive blood cultures without local inflammatory signs, those with no other source of infection, and those with a resolution of bacteremia after device extraction [9,22]. As there are no gold standard criteria for the diagnosis of device infection, the final diagnosis was established by the cardiology group according to the clinical, echocardiographic, and microbiologic findings [23].

Echocardiography was performed in all patients to establish the presence of endocardial or pacing lead vegetations in addition to valve involvement [21,24,25]. Lead vegetation was defined as the attachment of an oscillating or sessile mass to a lead associated with clinical manifestations of infection. Transthoracic echocardiography (TTE) was initially indicated in CDIE-suspected cases, and transesophageal echocardiography (TEE) was performed to more accurately visualize the intra- and extra-cardiac portions of the leads [26].

In the last few years, fluorine-18-marked fluorodesoxyglucose positron emission tomography and computed tomography (18F-FDG PET/CT) have also been included in the diagnosis of IE, as well as in the detection of extracardiac complications, according to the ESC guidelines [24].

After the CDIE diagnosis and blood cultures were taken, the patients were given empirical antibiotic therapy according to the ESC guidelines [24]. If the blood cultures were negative, the empirical therapy was continued, whereas in cases of positive blood culture results, pathogen susceptibility was established and antimicrobial treatment changed accordingly. In the majority of patients in our study, hardware extraction occurred within a week of initiating antibiotic therapy.

The primary outcome in this study was in-hospital mortality. Adverse events were collected at the time of hospital admission and during treatment until discharge from the hospital.

Baseline characteristics are summarized as the means ± standard deviation or medians and interquartile range (IQR) for continuous variables and frequencies with proportions for categorical variables, respectively. The differences between CDIE and left-sided IE patients were compared using the chi-square test or the Fisher exact test for categorical variables, and the Mann–Whitney U test or student’s *t*-test were used for continuous variables.

Logistic regression analyses were performed to determine the characteristics that were independently associated with in-hospital death, adjusted for age and sex. Baseline clinical, echocardiographic, and microbiological variables were tested as potential predictors of in-hospital mortality. Variables associated with death in the univariable analysis, as well as the well-known prognostic variables, were included in the multivariable logistic regression model. These included the presence of heart failure, chronic kidney disease, and embolic events at hospital admission, along with laboratory variables such as hemoglobin, leukocytes, and C-reactive protein levels. Additionally, *Staphylococcus aureus* as the causative pathogen, systemic embolization, and worsening heart failure were included in the final model. Calibration of the multivariable model was assessed by a goodness-of-fit test, and discrimination was measured by the area under the receiver operating characteristic (ROC) curve, or the *c* statistic. All analyses were performed using SPSS 20.0 (SPSS Inc., Chicago, IL, USA). Statistical significance was assumed at *p* < 0.05.

## 3. Results

### 3.1. Baseline Characteristics

Among the 274 patients admitted with IE, CDIE was diagnosed in 82 patients (30%), including 46 with a permanent pacemaker (56%), 23 with an ICD (28%), and 13 with a CRT device (16%). The clinical characteristics of CDIE compared with left-sided IE are shown in Table 1. The presence of the heart failure at diagnosis was the most commonly clinical presentation of CDIE, as shown in Table 2.

Local infection around the device, including pocket infection, was the most commonly clinical presentation of CDIE. Patients with CDIE were older with a high prevalence of diabetes mellitus compared with the other IE patients.

Evidence of systemic infection including fever, weight loss, anemia, and leukocytosis were detected to a lesser extent in CDIE than in left-sided IE. In the overall patient population, the classical signs of IE were infrequent, and they were particularly uncommon in patients with CRIE. Janeway lesions, Osler’s nodes, and splinter hemorrhages occurred in less than 4% of the cases. Splenomegaly was found in 3.8% of CDIE patients compared with 13% in left-sided IE patients (*p* = 0.023).

Of the eight embolic events experienced by patients with CDIE, seven were cases of pulmonary embolism.

The laboratory findings and the etiological agents identified in patients with cardiac-device-related endocarditisare shown in Table 2 and Table 3, respectively. Blood cultures were positive in 187 patients (68%), with 45 (55%) of the patients having CDIE. In contrast, 142 patients (74%) with left-sided IE had positive blood cultures (*p* = 0.001). The causal microorganisms isolated were predominantly staphylococci, specifically *S. aureus* in 25% of cases and coagulase-negative staphylococci in 29%. A positive culture for the typical causative agents from the pocket of the device or its leads was found in 20 patients (24%).

TEE was performed in 89% of the patients, whereas only TTE was used for the CDIE diagnosis in 11%. Vegetations were visualized in 74 patients (90%) by either TEE or TTE, of whom 88% had vegetation on a cardiac device lead. In three patients (4%), vegetation was questionable, and, in five patients (6%), no vegetation was detected. 18F-FDG PET/CT was performed in only three patients for the diagnosis of CDIE. Regarding the left ventricular ejection fraction (LVEF), the median LVEF in left-sided IE was 60 ± 16%, and the median LVEF in CDIE was 43 ± 13% (*p* < 0.001). The evidence suggests that patients with implant cardiac devices usually have more severe cardiomyopathies, especially those with ICD and CRT.

Concomitant native valve infection was found in 21 patients (26%) and prosthetic valve infection in three patients (3.7%). The distribution of valve involvement included the tricuspid (n = 7), mitral (n = 8), and aortic (n = 6) valves. Among the prosthetic valve infections, two patients had aortic and one had mitral prosthetic valve infections.

### 3.2. In-Hospital Outcomes

In the overall IE population, cardiac intervention, including surgery and device removal, was performed in 161 patients (59%). In 67 (82%) of those with CDIE, device and lead removal were performed after a week of antibiotic treatment. Fifteen patients (18%) did not undergo device removal during hospitalization due to unclear diagnoseis (including negative blood cultures, no vegetation by TEE, unknown source of bacteremia) and also reasons related to age, associated comorbid conditions, and technical issues. Moreover, 14 of the 21 patients with concomitant native valve infection (66.7%) underwent valve surgery during hospitalization, representing 17% of the overall CDIE patients.

The in-hospital outcomes are shown in Table 4. There was no difference in the mortality rate and other complications between the types of IE. Twenty-three patients with CDIE (28%) and 68 with left-sided IE (35%) died during hospitalization (*p* = 0.236). Regarding the device, mortality was higher among those with conventional pacemakers (52%) compared with ICD (43.5%) and CRT (4%, *p* < 0.05). The in-hospital mortality rate was similar in patients who underwent device removal and those who did not. Neurological complications and embolic events were similar between the groups (Table 4).

### 3.3. Predictors of Mortality in CDIE

In the overall population, the median duration of hospital stays for patients who survived was 46 days (IQR 32–56) compared with 25 days (IQR 15–54) for those who died (*p* <0.001). The hospitalization length was similar between CDIE and IE with no cardiac devices, with a median of 44.5 (IQR 30–56) and 41 (IQR 24–56), respectively. The leading cause of CDIE death was severe heart failure, which worsened during treatment. Table 5 presents the factors associated with in-hospital mortality in the subset of patients with CDIE.

Several factors were associated with in-hospital mortality in the setting of CDIE, including embolic events at diagnosis (Table 5). The inflammatory markers of disease severity, especially high white blood cell counts and CRP levels, were predictors of death. In the multivariable logistic regression, worsening of heart failure during the treatment was a strong predictor of in-hospital death (OR 3.105; 95% confidence interval [CI] 1.397–6.902). Additionally, CRP levels at diagnosis were independently associated with mortality (OR 1.006; 95% CI 1.001–1.1011). Removal of the cardiac device was not associated with lower in-hospital mortality.

The final multivariable model including *S. aureus* as an etiologic agent, worsening of heart failure, and CRP at diagnosis adjusted for age and sex showed optimal discrimination with a C statistic of 0.840 (95% CI 0.725–0.955) and demonstrated good calibration with a Hosmer–Lemeshow *p* value of 0.231.

## 4. Discussion

In this study, which included a large cohort of well-characterized spectra of IE cases over 10 years, we made important observations. First, among all the cases of IE, 30% were related to cardiac implantable devices. Second, the in-hospital mortality rate of CDIE was 28%, similar to other types of IE. Third, the CRP levels at hospital admission and worsening of heart failure during treatment were independent predictors of death in the setting of CDIE.

Cardiac-device-related infection is an uncommon but serious complication; it can manifest as an infection of the generator pocket and the leads, and it can also involve endocardial structures. This condition is associated with significant mortality [27,28] with an in-hospital mortality of 5–8% [16,29,30]. The mortality is mainly related to the complications of ongoing sepsis, but it is also influenced by comorbidities. In our study, the mortality was higher than in previously reported data, which was associated with underlying cardiac conditions. The majority of our patients with cardiac devices had heart failure, mainly due to Chagas disease, which is an important cause of pacemaker and ICD implantation in our setting. Our patients were selected from a tertiary care university hospital: a referral center with a high volume of device implantation that may explain the higher proportion of CDIE in our study population. Indeed, device-related infections constitute 10% of all endocarditis cases [1], which is a lower rate than was found in the present study.

Regarding the microorganisms identified in CDIE, we found a predominance of Gram-positive bacteria, particularly *S. aureus* and coagulase-negative staphylococci (38% of the cases), which are known for their affinity for prosthetic materials. The presence of these pathogens suggests that the etiology of device-related infections is closely linked to biofilm formation on the device surfaces, making treatment more challenging and highlighting the importance of preventive strategies in managing these infections.

Previous studies in this field have focused on the risk of device infection and the subsequent IE following implantation of a cardiac electronic device [8,9,11,27,28,31]. Also, these studies reported the mortality rates of CDIE compared with non-infected patients [8,14,16,30]. On the other hand, in our study, we examined the risk factors for mortality in an overall CDIE population compared with IE but with no cardiac device in place. Our aim was not to assess the risk of IE in patients with cardiac devices, which has been extensively studied.

The mortality rate in CDIE is high in patients with significant comorbidities, especially heart failure, diabetes, and renal failure, which is in agreement with our findings. Indeed, many deaths are not infection-related [26]. In-hospital morbidity may be related to complications of lead extraction, including emergency thoracotomy for perforation, tricuspid valve damage, septic pulmonary emboli, arrhythmias, and ongoing sepsis [32]. Following device removal, complications can arise from the re-implantation of a new device, particularly recurrent infection, although it is uncommon provided appropriate antibiotic treatment has been undertaken and the timing of the new implant is correct [16].

However, it is not clear whether the higher mortality is due to the CDIE itself, due to the presence of poor prognostic factors in the patients who develop CDIE, or whether a poor outcome reflects inadequate management. There is some evidence that patients who have been successfully treated with a complete removal of hardware and a full course of antibiotics may have a similar prognosis to patients who have never been infected [16,33,34]. In the EURO-ENDO registry, which includes centers worldwide, device removal was identified as a critical factor for improving prognosis, regardless of the pathogen involved [15]. When device removal was recommended but could not be completed due to the patient’s condition or technical concerns, it became a significant risk factor for mortality. Additionally, lead extraction should also be considered, and not only when a pre-operative CDIE is diagnosed. Notably, even in cases of left heart IE with an apparently non-infected cardiac device, it is crucial to treat the IE and remove the intracardiac device. In contrast, in the present study, the mortality rate was similar between the patients who underwent device extraction and those who did not.

The in-hospital mortality rate for isolated CDIE in the EURO-ENDO registry was lower than in the present study (13% versus 28%) [15]. The majority of our patients with cardiac devices had heart failure, mainly due to Chagas disease. This condition, which is less common in the EURO-ENDO registry, requires frequent reimplantation of the cardiac device after the removal of the infected one due to severe arrhythmias. It was demonstrated that cardiac device removal/reimplantation is a factor associated with increased one-year mortality in CDIE [35]. Furthermore, according to a publication from the EURO-ENDO registry, heart failure was one of the factors independently associated with mortality (hazard ratio [HR] 1.64), and our patients had a high rate of heart failure with frequent tricuspid regurgitation and important impairments of right ventricular function. In this context, it has also been demonstrated that moderate or severe tricuspid regurgitation (HR 4.24) and abnormal right ventricular function (HR 3.59) are independent factors associated with six-month mortality in CDIE [16]. In the multivariable logistic regression of our study, worsening of heart failure during the treatment was a strong predictor of in-hospital death (OR 3.105).

The inflammatory markers of infection severity, especially CRP levels at diagnosis, were an independent predictor of death in CDIE. Most studies assessing CRP in the setting of IE demonstrated its value in the diagnosis of the disease [20,36,37]. An elevated level of this marker supports the diagnosis of IE, whereas normal levels indicate a low probability of this condition. Consequently, some investigators have suggested the inclusion of CRP as a minor Duke criterion for the diagnosis of IE [37]. Regarding prognostic implications of CRP, scarce data are available. In certain studies, serial CRP measurements for the prediction of outcomes were used [36,38].

This study has some limitations. Although TEE images more accurately demonstrate leads, valvular involvement, and vegetation, echocardiography cannot discriminate infective vegetation from non-infected thrombotic or fibrous masses, which are also prevalent on chronic indwelling leads [35]. Therefore, some cases of CDIE may have been missed from our study. Early on post-operatively, it may be difficult to differentiate between CDIE pocket infection and superficial wound infection. Diagnostic percutaneous punctures with pocket fluid aspiration should be avoided [12]. Although multi-modality imaging is recommended for improving diagnosis, only a few patients underwent 18F-FDG PET/CT for the diagnosis of CDIE in our study. It is crucial to identify patient groups that would benefit most from advanced imaging techniques, which requires further investigation. The high prevalence of Chagas disease in our cohort limited the generalization of the results. Additionally, this was a single-center study from a large tertiary hospital; thus, it has inherent selection biases, thereby limiting its generalizability. This study included a large cohort of IE, and cardiac CDIE constituted 30% of all endocarditis cases over a 10-year period with an in-hospital mortality rate of 28%. The predictors of mortality were high CRP levels at hospital admission and worsening of heart failure during treatment. CDIE is increasingly recognized as a serious and potentially lethal condition, and it is often challenging to diagnose and requires high clinical suspicion. Further investigations are needed to define the optimal timing for device removal and to evaluate long-term outcomes beyond the immediate in-hospital period.

## Figures and Tables

**Table 1:** Baseline characteristics of the study population stratified according to the type of infective endocarditis.

Variables	Left-Sided IE * (n = 192)	Device-Related IE (n = 82)	*p*-Value
Baseline characteristics			
Male sex	113 (58.9)	53 (64.6)	0.370
Age (years)	47.5 ± 16.4	55.8 ± 16.4	<0.001
Predisposing conditions			
Rheumatic heart disease	73 (38.0)	10 (12.2)	<0.001
Degenerative valve disease	25 (13.4)	4 (4.9)	0.110
Mitral valve prolapse	24 (12.8)	5 (6.1)	0.125
Valve prosthesis	63 (33.0)	8 (10.1)	<0.001
Congenital heart disease	15 (7.9)	8 (9.8)	0.375
Previous IE	19 (10.1)	6 (7.3)	0.467
Diabetes mellitus	21 (11.1)	19 (23.2)	0.010
Use of immunosuppressants	15 (7.9)	8 (9.9)	0.582
Chronic kidney disease	29 (15.3)	6 (7.3)	0.076
Central IV access	30 (15.9)	7 (8.5)	0.106
Bacteremia source identified	77 (40.2)	41 (50.0)	0.327
Manipulation of the oral cavity	33 (17.3)	5 (5.9)	0.023
Skin manipulation	16 (8.3)	6 (7.4)	0.804
Operative wound/pocket infection	17 (8.9)	23 (27.9)	<0.001
Previous antibiotic use	76 (39.9)	33 (41.0)	0.804

Data are expressed as the mean value ± standard deviation or as absolute numbers (percentage) of patients. * defined as native-valve infective endocarditis or as prosthetic-valve endocarditis. IE: infective endocarditis; IV: intravenous.

**Table 2:** Clinical manifestations, laboratory findings, main causative microorganisms, and echocardiographic features stratified according to type of infective endocarditis.

Variables	Left-Sided IE (n = 192)	Device-Related IE (n = 82)	*p*-Value
Clinical manifestations			
Anorexia/hyporexia	113 (59.2)	36 (44.4)	0.026
Fever	161 (84.0)	49 (60.5)	<0.001
Weight loss	98 (51.1)	24 (29.1)	0.001
Sweating	57 (29.8)	21 (26.3)	0.835
Neurological event	39 (20.7)	8 (9.9)	0.033
Embolic event	27 (14.2)	8 (10.1)	0.370
Skeletal muscle manifestation	36 (18.7)	5 (6.3)	0.032
Heart murmur	152 (79.1)	31 (37.5)	<0.001
Splenomegaly	25 (13.0)	3 (3.8)	0.023
Heart failure at diagnosis	111 (58.2)	55 (67.9)	0.134
Chagas disease	2 (1)	23 (28)	<0.001
Laboratory findings			
Anemia	161 (84.1)	58 (71.6)	0.018
Leukocytosis	112 (58.7)	33 (40.0)	0.005
CRP (mg/L)	76 [35/181]	63 [20–161]	0.274
Main causative microorganisms *			
Positive blood culture	142 (74.0)	45 (54.9)	0.001
*Staphylococcus aureus*	42 (21.9)	15 (18.3)	0.661
Coagulase-negative staphylococci	21 (13.0)	16 (19.5)	0.025
Streptococci	34 (21.0)	2 (3.2)	0.001
*Enterococcus* sp.	20 (10.4)	1 (1.2)	0.003
Gram-negative bacteria	5 (2.6)	7 (8.5)	0.001
Echocardiographic features			
Presence of vegetation	158 (82.3)	74 (90.1)	0.231
Vegetation length (mm)	12.96 ± 8.0	12.98 ± 6.6	0.982

Data are expressed as the mean value ± standard deviation, median [interquartile range], or absolute numbers (percentage) of patients. CRP: C-reactive protein. * defined as negative blood cultures or unidentified microorganisms, and these were observed in 87 patients (31.7% of the total).

**Table 3:** The etiological agents identified in patients with cardiac-device-related endocarditis.

Etiologic Agent	Device-Related IE with Positive Blood Culture (n = 45)
*Staphylococcus aureus*	15 (18.3%)
MSSA	9
MRSA	6
Coagulase-negative staphylococci	16 (19.5%)
*Staphylococcus epidermidis*	11
*Staphylococcus hominis*	4
*Staphylococcus warneri*	1
Viridans streptococci	2 (2.4%)
*Streptococcus mitis*	1
*Streptococcus oralis*	1
*Enterococcus* sp.	1 (1.2%)
Gram-negative bacteria	7 (8.5%)
*Klebsiela pneumoniae*	2
*Serratia marcescens*	2
*Pseudomonas aeruginosa*	2
*Enterobacter aerogenes*	1
HACEK	2 (2.4%)
*Aggregatibacter actinomycetemcomitans*	1
*Aggregatibacter* sp.	1
Fungi	1 (1.2%)
*Candida* sp.	1
Others	1 (1.2%)

MSSA: methicillin-susceptible *S. aureus*; MRSA: methicillin-resistant *S. aureus*; HACEK: *Haemophilus aphrophilus* (subsequently called *Aggregatibacter aphrophilus* and *Aggregatibacter paraphrophilus*), *Actinobacillus actinomycetemcomitans* (subsequently called *Aggregatibacter actinomycetemcomitans*), *Cardiobacterium hominis*, Eikenella corrodens, and Kingella kingae.

**Table 4:** In-hospital outcomes stratified according to type of infective endocarditis.

Variables	Left-Sided IE (n = 192)	Device-Related IE (n = 82)	*p*-Value
In-hospital death	68 (35.4)	23 (28.0)	0.236
Cardiac interventions *	94 (49.2)	67 (81.7)	<0.001
Cardiac complications	72 (37.8)	27 (32.9)	0.496
Neurological complications	27 (14.1)	7 (8.5)	0.233
Systemic embolization during treatment	24 (12.5)	7 (8.5)	0.364
Prolonged fever > 10 days	38 (19.8)	9 (11.0)	0.129

Data are expressed as the absolute numbers (percentage) of patients. * Cardiac interventions: cardiac surgery or complete hardware removal (device and lead extraction).

**Table 5:** Predictors of in-hospital mortality in patients with device-related infective endocarditis (n = 82).

Covariates		Odds Ratio	(95% CI)	*p*-Value
Univariable analysis				
Data at diagnosis	Age (years)	1.002	0.973–1.032	0.893
	Male sex	1.254	0.463–3.394	0.656
	Prior heart failure	2.903	0.873–9.653	0.082
	Chronic kidney disease	0.919	0.213–3.324	0.806
	Embolic events	9.529	1.757–51.675	0.009
	Hemoglobin (g/dl)	0.711	0.544–0.929	0.012
	Leukocytes (cells × 10^3^/µL)	1.088	1.022–1.158	0.008
	C-reactive protein (mg/L)	1.004	1.000–1.008	0.028
	*Staphylococcus aureus*	0.360	0.065–1.985	0.241
During treatment	Heart failure *	3.281	1.695–6.351	<0.001
	Systemic embolization	0.513	0.105–2.498	0.408
Multivariable analysis				
C-reactive protein (mg/L)		1.006	1.001–1.011	0.016
Worsening of heart failure		3.105	1.397–6.902	0.005

* Heart failure: stable vs. refractory. The model included *Staphylococcus aureus* as an etiologic agent, worsening of heart failure, and the C-reactive proteins at diagnosis, and these were adjusted for age and sex, showing optimal discrimination with a C statistic of 0.840 (95% CI 0.725–0.955) and demonstrating good calibration with a Hosmer–Lemeshow *p* value of 0.231. CI: confidence interval.

## Data Availability

The datasets presented in this article are not readily available due to privacy and ethical reasons. Requests to access the datasets should be directed to M.C.P.N. (mcarmo@waymail.com.br).
